# When More Transmission Equals Less Disease: Reconciling the Disconnect between Disease Hotspots and Parasite Transmission

**DOI:** 10.1371/journal.pone.0061501

**Published:** 2013-04-08

**Authors:** Andrew W. Park, Krisztian Magori, Brad A. White, David E. Stallknecht

**Affiliations:** 1 Odum School of Ecology, University of Georgia, Athens, Georgia, United States of America; 2 Department of Infectious Diseases, College of Veterinary Medicine, University of Georgia, Athens, Georgia, United States of America; 3 Southeastern Cooperative Wildlife Disease Study, College of Veterinary Medicine, University of Georgia, Athens, Georgia, United States of America; University of California, Berkeley, United States of America

## Abstract

The assumed straightforward connection between transmission intensity and disease occurrence impacts surveillance and control efforts along with statistical methodology, including parameter inference and niche modeling. Many infectious disease systems have the potential for this connection to be more complicated–although demonstrating this in any given disease system has remained elusive. Hemorrhagic disease (HD) is one of the most important diseases of white-tailed deer and is caused by viruses in the *Orbivirus* genus. Like many infectious diseases, the probability or severity of disease increases with age (after loss of maternal antibodies) and the probability of disease is lower upon re-infection compared to first infection (based on cross-immunity between virus strains). These broad criteria generate a prediction that disease occurrence is maximized at intermediate levels of transmission intensity. Using published US field data, we first fit a statistical model to predict disease occurrence as a function of seroprevalence (a proxy for transmission intensity), demonstrating that states with intermediate seroprevalence have the highest level of case reporting. We subsequently introduce an independently parameterized mechanistic model supporting the theory that high case reporting should come from areas with intermediate levels of transmission. This is the first rigorous demonstration of this phenomenon and illustrates that variation in transmission rate (e.g. along an ecologically-controlled transmission gradient) can create cryptic refuges for infectious diseases.

## Introduction

Positive correlation between the amount of parasite transmission and the abundance or probability of disease is both intuitive and commonly observed. However, there are easily-satisfied conditions that generate more complex predictions. Enzootic or endemic stability refers to systems where the occurrence of symptomatic cases is maximized at intermediate levels of transmission [1], [2]. Coleman et al. [2] outlined two sufficient criteria for this to occur: that (1) disease is more likely (or severe) in older individuals and (2) initial infection reduces the probability of subsequent infection (or manifestation of symptoms in cases of re-infection). Because these criteria are general and independent of specific mechanisms, they are thought to be satisfied in disease systems spanning human and animal hosts; viral, bacterial and protozoan parasites; and direct and vector-borne transmission mechanisms [2]. However, bringing together compelling evidence of the manifestation of this disconnection between transmission intensity and disease occurrence has proved elusive.

The genus *Orbivirus* contains over a hundred viral serotypes [3] that are predominantly vectored by biting midge species in the genus *Culicoides* [4], and have been implicated in human, domestic animal and wildlife diseases [5]. Within this genus multiple serotypes of viruses in the closely-related bluetongue (BTV) and epizootic hemorrhagic disease (EHDV) serogroups have been associated with significant disease in white-tailed deer (WTD, *Odocoileus virginianus*) populations [5]. Because the diseases caused by EHDV (EHDV-1 and -2) and BTV (BTV-10, -11, -13, and -17) in WTD are clinically indistinguishable they are collectively referred to as hemorrhagic disease (HD) [6]. In the US, HD also occurs in mule deer, *Odocoileus hemionus* [7] but the abundance and range of WTD renders them the more important species of the two. Deer can survive infection and in cases of morbidity, clinical manifestation includes sloughing hooves, ulcers in the mouth and scars on the rumen lining [5]. The HD system satisfies enzootic stability via specific processes. Regarding requirement that disease is more likely in younger animals, deer fawns born in spring of previously exposed does have maternal antibodies, which last for up to 18 weeks [8], well into the activity season of *Culicoides* vectors. Consequently disease is relatively less likely compared to the does themselves who have only one line of protection, infection-induced antibodies that can wane within a year causing adults to revert to a susceptible status [9]. The second requirement (that re-infection is less likely to cause symptoms than a first infection) is satisfied by the observation that deer previously infected with virus show no or minimal symptoms on experimental reinfection with related virus in contrast to naïve deer, which show severe clinical disease following the same experimental infection [10]. However, deer infected with a second serotype have similar viral titer profiles to those observed during primary infection, indicating their capacity for asymptomatic transmission [10]. The presence of heterologous virus is important because a seropositive status to homologous virus challenge leads to both protection from symptoms and lack of viremia in this system [11]. Whether the heterology required to generate asymptomatic infection needs to be as extreme as distinct serotypes (versus antigenic variation within a serotype) remains an open question for the HD system. In any case, multiple serotypes (especially EHDV-1 and -2) routinely co-circulate in the regions of study presented here.

A series of state-level studies of WTD populations in the US [12]–[15] suggests that there is considerable variation in transmission, with reported mean seroprevalence values in the range 8–84%. Seroprevalence determines the proportion of individuals in a population that have current or previous exposure to virus by testing for antibodies. High levels of seroprevalence are therefore indicative of high transmission levels. Each of the four published studies was aimed at obtaining an objective measure of seroprevalence in a state. Geographical sampling was well dispersed and generally included the whole WTD range. Each study was also multi-year (range 2–9 years) and not in response to a particular outbreak. Surveillance data coordinated by the Southeastern Cooperative Wildlife Disease Study (SCWDS) has recorded presence/absence of HD mortality and morbidity in WTD at the county level confirmed by state vets over ∼30 years. Morbidity records are based on observation of hunter-killed deer that showed sloughing hooves, ulcers in the mouth or scars on the rumen lining. Mortality is based on fulfillment of either: (1) sudden, unexplained high deer mortality during the late summer and early fall; (2) necropsy diagnosis of HD obtained by a trained wildlife biologist, a diagnostician at a State Diagnostic Laboratory or Veterinary College, or by SCWDS personnel; (3) isolation of EHDV or BTV from a deer.

Viral isolation has confirmed that the vast majority of HD cases (∼90%) are associated with EHDV and that both serotypes (EHDV-1 and -2) routinely co-circulate in the regions described here. Based on the seroprevalence and disease reporting data, we use a statistical model to predict disease occurrence as a function of seroprevalence. Additionally, we parameterize a mechanistic transmission model for this system, an age-structured variant of the SIR model [16], to generate predictions of how transmission rate affects seroprevalence and the number of symptomatic cases. A key component of the model is the co-circulation of two-serotypes. The occurrence of asymptomatic infection in the HD system, which is central to the concept of enzootic stability, relates to consecutive infection by viral strains from distinct serogroups. Collectively, these analyses provide evidence for enzootic stability. Finally, we discuss the impact of a disconnection between transmission intensity and disease hotspots on explaining, predicting, surveying and controlling infectious diseases.

## Materials and Methods

Published seroprevalence studies of BTV and EHDV in WTD [12]–[15] were collated at the state level for each of the 16 states sampled (Alabama, Arkansas, Florida, Georgia, Kansas, Kentucky, Louisiana, Maryland, Mississippi, North Carolina, Oklahoma, South Carolina, Tennessee, Texas, Virginia, West Virginia–detailed in Table 1). Although the seroprevalence protocol was not identical between these studies, they were all aimed at establishing baseline seroprevalence values in different geographic regions, and within a region considerable effort was made to survey objectively in the complete range of WTD.

**Table 1:** Studies estimating seroprevalence and case prevalence of HD in various US states (case prevalence is calculated from Southeastern Cooperative Wildlife Disease Study records 1980–2007).

State	Number positive	Number sampled	Percent positive	Virus	Study years	Reference	County-years positive	County-years reporting	Proportion positive
	Seroprevalence						Case prevalence		
Alabama	70	140	50	EHDV, BTV or Both	1981–1989	[13]	421	1876	0.22
Arkansas	56	164	34	EHDV, BTV or Both	1981–1989	[13]	293	2100	0.14
Florida	95	189	50	EHDV, BTV or Both	1981–1989	[13]	155	1876	0.08
Kentucky	4	21	19	EHDV, BTV or Both	1981–1989	[13]	286	3360	0.09
Louisiana	61	164	37	EHDV, BTV or Both	1981–1989	[13]	200	1792	0.11
Maryland	6	68	9	EHDV, BTV or Both	1981–1989	[13]	75	672	0.11
Mississippi	27	57	47	EHDV, BTV or Both	1981–1989	[13]	703	2296	0.31
North Carolina	4	44	9	EHDV, BTV or Both	1981–1989	[13]	333	2800	0.12
South Carolina	65	112	58	EHDV, BTV or Both	1981–1989	[13]	237	1288	0.18
Tennessee	11	29	38	EHDV, BTV or Both	1981–1989	[13]	173	2660	0.07
Virginia	2	25	8	EHDV, BTV or Both	1981–1989	[13]	809	3752	0.22
West Virginia	31	125	25	EHDV, BTV or Both	1981–1989	[13]	74	1540	0.05
Georgia	699	3077	23	EHDV, BTV or Both	1981–1989	[13]	599	4452	0.13
Kansas	60	87	69	EHDV, BTV or Both	1998–2002	[15]	79	2940	0.03
Oklahoma	146	194	75	EHDV or BTV	1977–1984	[12]	29	2156	0.01
Texas	574	685	84	EHDV, BTV or Both	1991–1992	[14]	108	7112	0.02

SCWDS records of presence/absence of HD-related WTD mortality and morbidity at the county level for the corresponding states (1980–2007) were used to estimate likelihood of disease reporting. Surveillance data on HD was aggregated at the state level. For a state containing *n* counties, ‘proportion of county-years reporting’ was defined as the sum of the number of years (maximum 28) each county belonging to a target state reported HD morbidity or mortality divided by 28*n*. A loess smoothing model [17], a non-parametric regression model, of the raw presence/absence data was used to obtain a prediction of this metric as a function of seroprevalence, using a span of 0.8.

In regions where seroprevalence data was collected, HD-related morbidity is more commonly reported than mortality. Accordingly, a mechanistic transmission model was constructed to reflect the HD biology in these regions. The host population (total size *N*) was divided into two age-classes, fawns and does. Bucks were not included explicitly in the model. Fawns matured into does, and does gave birth to fawns at a constant rate. For does, four epidemiological states were possible in relation to a viral serotype: susceptible (*S*), symptomatically infectious (*I*), asymptomatically infectious (*A*), and recovered (*R*). Fawns had an additional status (*P*) meaning they had received protection from maternally-acquired antibodies. All individuals in the population were assigned to one class based on their status in relation to two co-circulating serotypes (nominally EHDV-1 and -2). For example the [*AP*]*_F_* class contained fawns that were asymptomatically infected with serotype-1 and carrying maternal antibodies from their mother's infection by serotype-2. Similarly, the [*SI*]*_D_* class contained does that were susceptible to serotype-1 and infectious with serotype-2. Accounting for the possible change in states, the full model yielded 21 equations for the fawns and 14 equations for the does. However, in spite of its size the model is closely related to standard compartment models such as the SIR model, and was well parameterized from data (Table 2).

**Table 2:** Model parameters used in the mechanistic system of ordinary differential equations.

Parameter	Definition	Default value	Reference
*λ*	Birth rate	0.00216 days^−1^	[30]
*μ*	Mortality rate	0.00063 days^−1^	[30]
*δ*	Maturation rate	0.00274 days^−1^	[31]
*β*	Transmission rate	0.01–100 days^−1^	Varied
*γ*	Recovery rate	0.0166 days^−1^	[10]
*ω*	Rate of waning immunity	0.00274 days^−1^	[9] Varied in Figures S1 & S2
*σ*	Rate of waning maternal antibodies	0.00794 days^−1^	[8]

**Figure 1:**
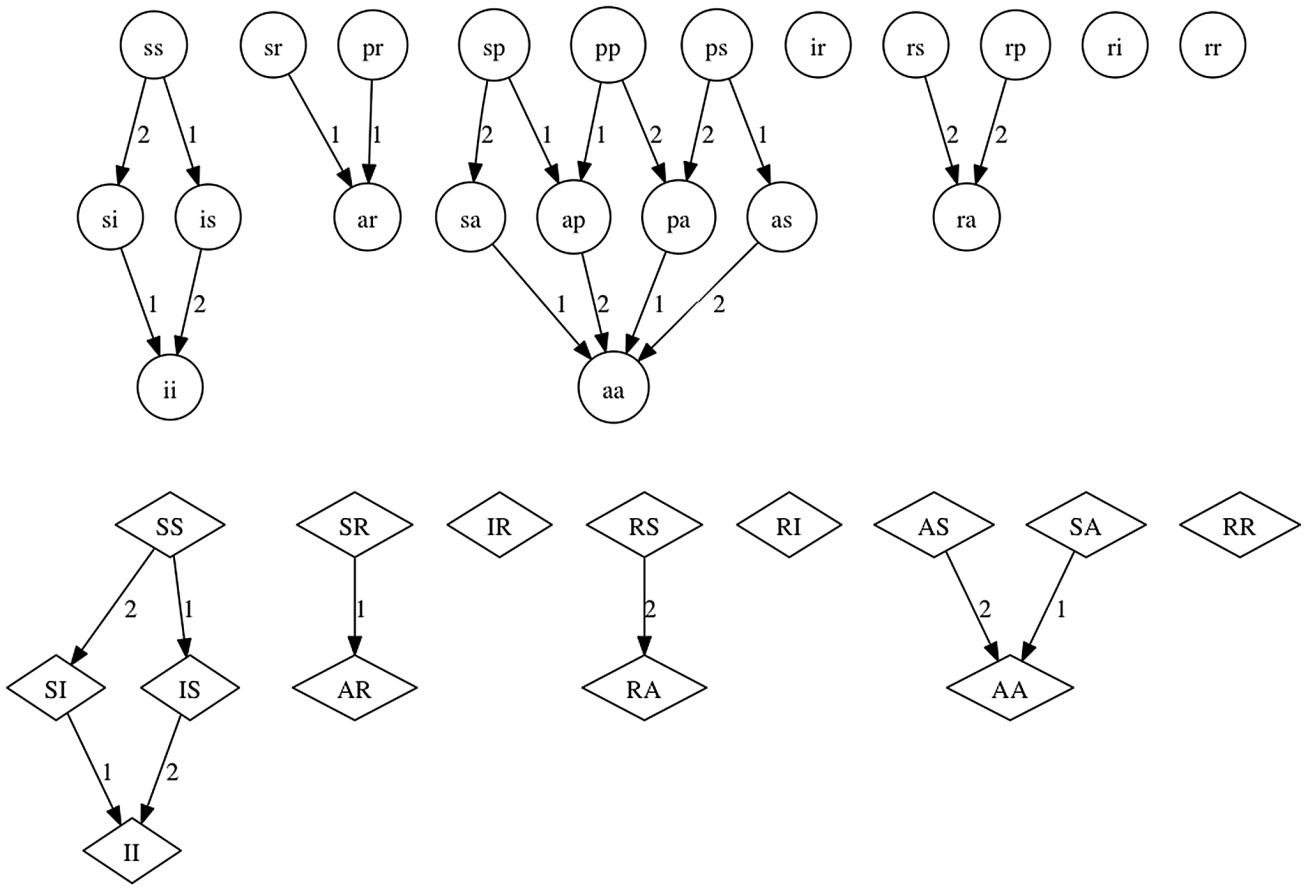
Transmission graphs for the mechanistic model. Each fawn class is represented by a circle with two small case letters giving the status with respect to serotypes 1 and 2. Similarly, doe classes are represented by diamonds with two large case letters. Arrows represent possible changes of state due to infection events. Arrows are numbered 1 and 2 to represent the infecting serotype.

**Figure 2:**
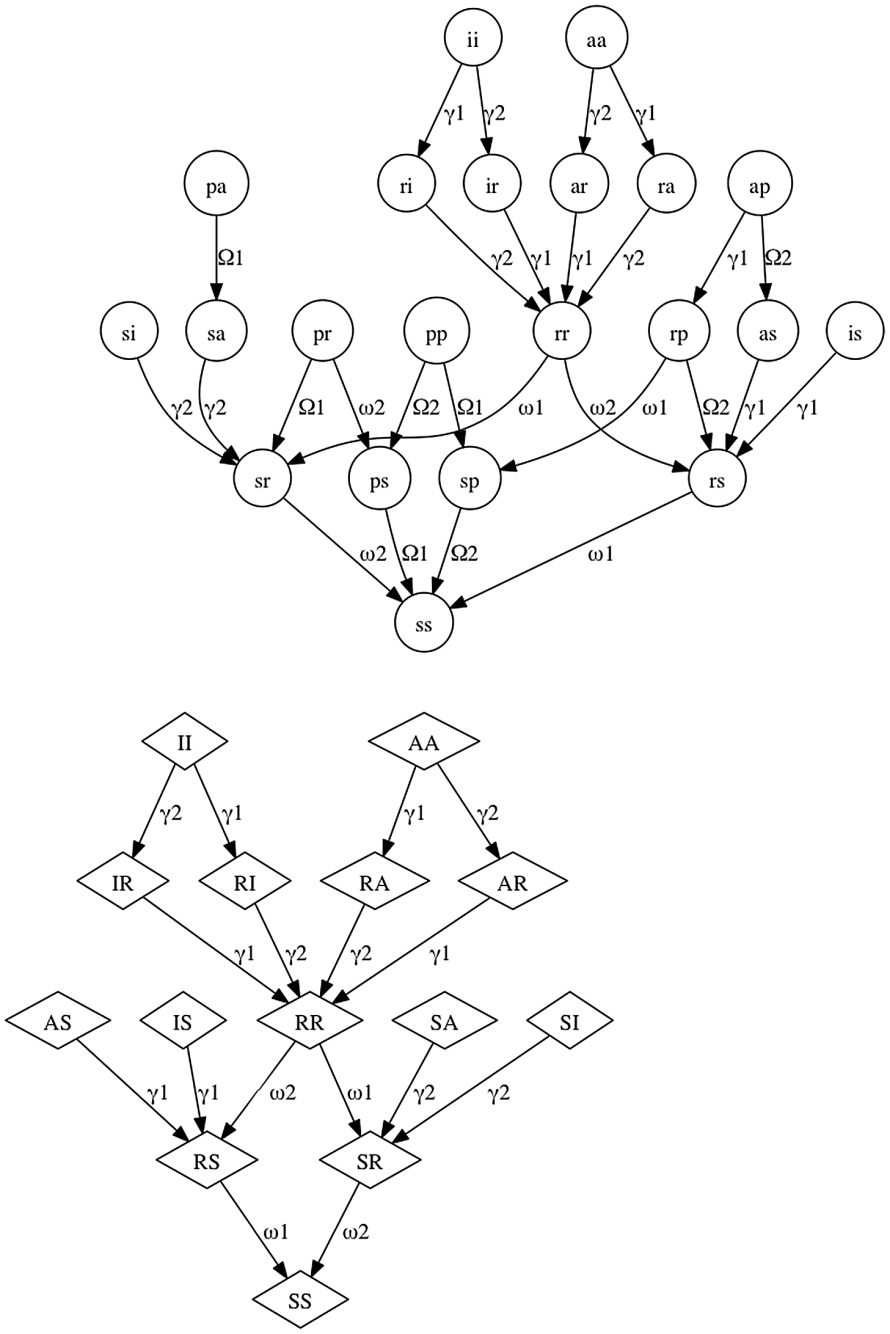
Recovery/waning graphs for the mechanistic model. Fawn and doe classes are as in Figure 1. Arrows indicate antibody-mediated recovery from symptomatic or asymptomatic infection (labeled γ), or waning of disease-induced (labeled ω) and maternally acquired (labeled Ω) antibodies. Numbers on arrows represent the viral serotype associated with the antibodies.

**Figure 3:**
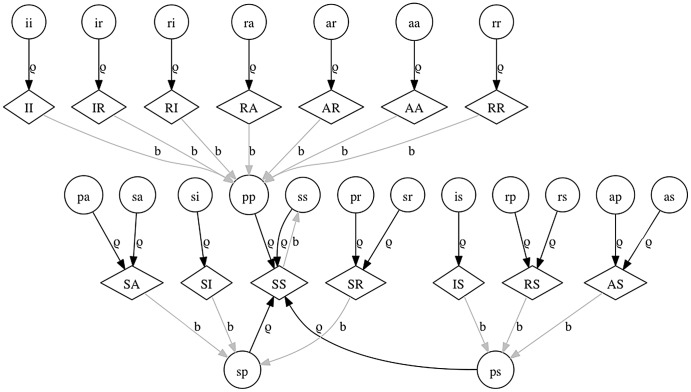
Births and aging graph for the mechanistic model. Fawn and doe classes are as in Figure 1. Black arrows (labeled ρ) indicate the transitions associated with aging of fawns to does. Grey arrows (labeled b) indicate type of fawn offspring generated by each doe class.

The full model equations are provided as (Equations S1), and figures 1, 2, 3 show the flows in the model relating to transmission (Fig. 1), recovery and waning (Fig. 2), and births and aging (Fig. 3). Natural mortality also occurs in the model. Transmission was modeled as frequency-dependent and any class susceptible to one of the serotypes experienced a force of infection composed of all states infectious with that serotype. Therefore both symptomatic and asymptomatic fawns and does contributed to the transmission process equally. Likewise, the duration of subsequent (versus initial) infections was assumed to be equal. This is in agreement with viral dynamics measured in challenge experiments [8].

Animals in epidemiological states *A*, *R* or *P* regarding one serotype, but susceptible to the second, could only become asymptomatically infectious with the second serotype. Correspondingly, animals in epidemiological states *S* or *I* became symptomatically infectious with the second serotype. This is based on experimental reinfection data [10], where the presence of preexisting antibodies for one serotype lead to an asymptomatically infectious status for the second serotype upon reinfection.

Waning occurred on a per-serotype basis whereby animals could revert to an *S*-state with respect to one serotype but retain their independent state regarding the other serotype (e.g. [*RP*]*_F_* could revert to [*SP*]*_F_*). This assumption is based on data for the reverse (infection) process where antibodies are not cross-protective regarding infection, rather they protect against symptomatic infection [10]. Regarding aging, fawn classes matured into equivalent doe classes with the exception that any *P*-status fawns matured into corresponding *S*-status does, consistent with maternal antibodies waning by the time an animal reaches adult status [9].

The model did not explicitly include the vector population, for which data is scarce. Rather, transmission was approximated as direct between hosts, as can be done to avoid introducing complexity without suitable parameter estimation [18]. The system of ordinary differential equations was integrated over a 28-year period (reflecting the time-span of data collection) for a range of transmission rates, and the cumulative incidence (total number of symptomatic cases) and seroprevalence (proportion of the population infected, sampled towards the end of the simulation) were recorded. The model was initialized with all states equal to zero except [*SS*]*_F_* = 10^5^−2, [*SI*]*_F_* = 1, [*IS*]_F_ = 1. Exploratory analysis showed that results were insensitive to a range of plausible initial conditions provided both serotypes were represented.

Sampling from the model was done in order to generate predictions that are more closely related to the data. Available data relates seroprevalence to the proportion of county-years reporting, whereas the model relates transmission rates to both seroprevalence and proportion of county-years reporting. Accordingly, 100 samples of the model were performed per transmission rate. The range of transmission rates was restricted to between *β* = 10^−1.4^ and *β* = 10^−0.4^ (log scale, step size = 10^−0.2^) as this range spans the observed seroprevalence values in the data. Based on equilibria for model states, the probabilities of an animal being seropositive and of being symptomatically infected were calculated. For each sampling, a binomial trial was performed 100 times (reflecting 100 animals), with the relevant probability (seropositive or symptomatically infected). Ultimately, a further subsampling was performed to ensure that 50 data points were used per seroprevalence range (20–40%, 40–60%, 60–80%, 80–100%) in plotting the relationship. This was done to avoid the high seroprevalence bias introduced by varying the transmission rate on a log scale.

The duration of protection in WTD following natural infection is difficult to establish with certainty. In Stallknecht et al. [9], a WTD cohort on a barrier island was tracked over 6 years following an outbreak. The number of seropositive animals was observed to drop each year, and between some years as many as 30% of animals would revert to seronegative status. Consequently, in the main mechanistic model we selected a mean duration of infection of 1 year. However, although the data may support a declining “detectable” antibody response, this may not accurately represent protection status, especially if there is a significant cell mediated response, which is common with viruses. Additionally, it is impossible to rule out the possibility of re-exposures (natural boosting). Consequently, we extended our analysis to evaluate model predictions under scenarios of (i) a relatively short duration of protection (6 months–interpreting the presence of antibodies as evidence of re-exposure) and (ii) a relatively long duration (mean life expectancy–essentially assuming life-long protection involving cell mediated immunity not captured in the data).

## Results

**Figure 4:**
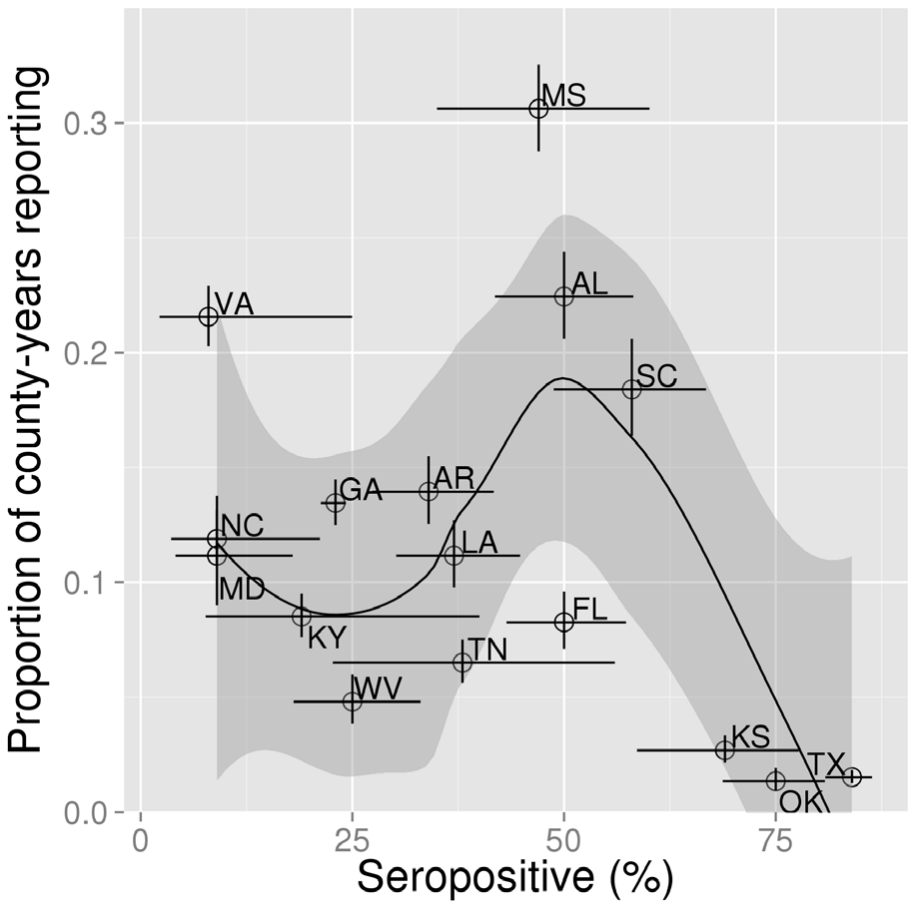
Amount of case reporting as a function of seroprevalence. Proportion of 'county-years' reporting morbidity and/or mortality of HD as a function of seroprevalence in 16 US states. Open circles represent mean values. Error bars are 95% binomial confidence intervals. A loess model (span = 0.8) fitted to these data is also shown (solid black line). The shaded area around the fitted line is the 95% CI based on the standard errors of the locally weighted least squares regression using the t-based approximation.

There is strong evidence that case reporting is maximized at intermediate levels of seroprevalence (Fig. 4). A smoothing function (loess, span = 0.8) fitted to the data shows that case reporting is maximized at ∼50% seroprevalence (Fig. 4–solid black line). This simple statistical model provides an objective assessment of the high case reporting at intermediate levels of seroprevalence, as well as the low case reporting associated with high levels of seroprevalence. Confidence intervals (Fig. 4–shaded area around fitted line) lend further support to the non-monotonic, unimodal nature of the relationship.

**Figure 5:**
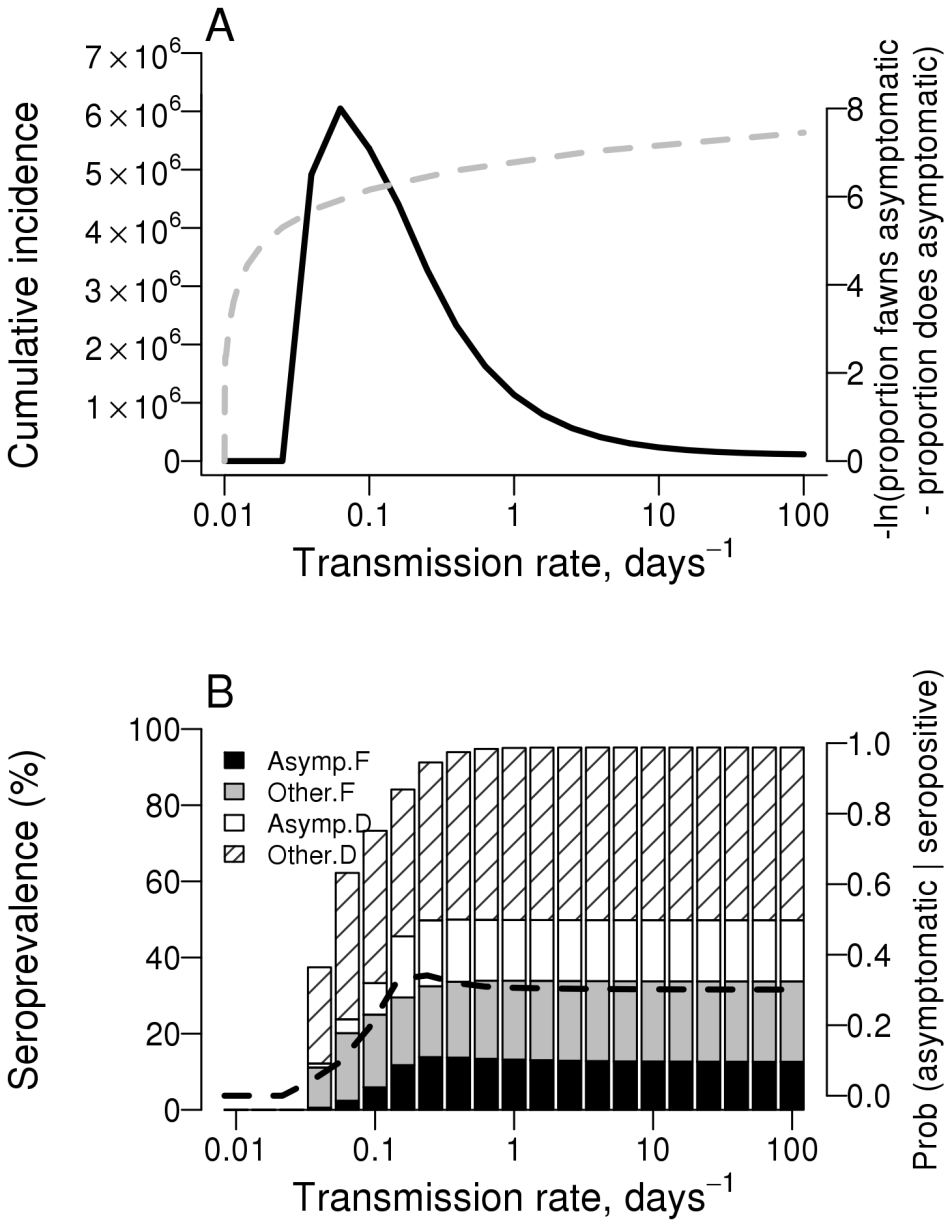
Model predictions for cumulative incidence and seroprevalence as a function of transmission intensity. A: cumulative incidence (total number of symptomatic cases) over a 28-year simulation as a function of various transmission rates. B: Seroprevalence as a function of transmission rate and stratified by infected subpopulation: black bars  =  asymptomatically infected fawns, gray bars  =  all other seropositive fawns, white bars  =  asymptomatically infected does and striped bars  =  all other seropositive does. The dashed black line corresponds to the second y-axis and indicates the probability of currently being asymptomatically infected given a seropositive status. Default parameters as in Table 2.

The mechanistic model describing transmission in the HD system shows that cumulative incidence (total number of symptomatic cases) is initially expected to increase with transmission rate and then decline towards zero at high transmission rates (Fig. 5A–black solid line). The model also predicts that disease is more likely (per infection) in older than younger animals (Fig. 5A–gray dashed line). In contrast to the unimodal relationship predicted between transmission rate and cumulative incidence, seroprevalence is expected to monotonically increase with transmission rate (Fig. 5B). At relatively low transmission rates, seropositive samples are expected to be largely associated with symptomatic deer (Fig. 5B-hatched and gray components of bar plot, also black dashed line) whereas at high transmission rates the association weakens with a substantial proportion (∼1/3) of seropositive cases relating to active asymptomatic infection (Fig. 5B–black and white components of bar plot).

**Figure 6:**
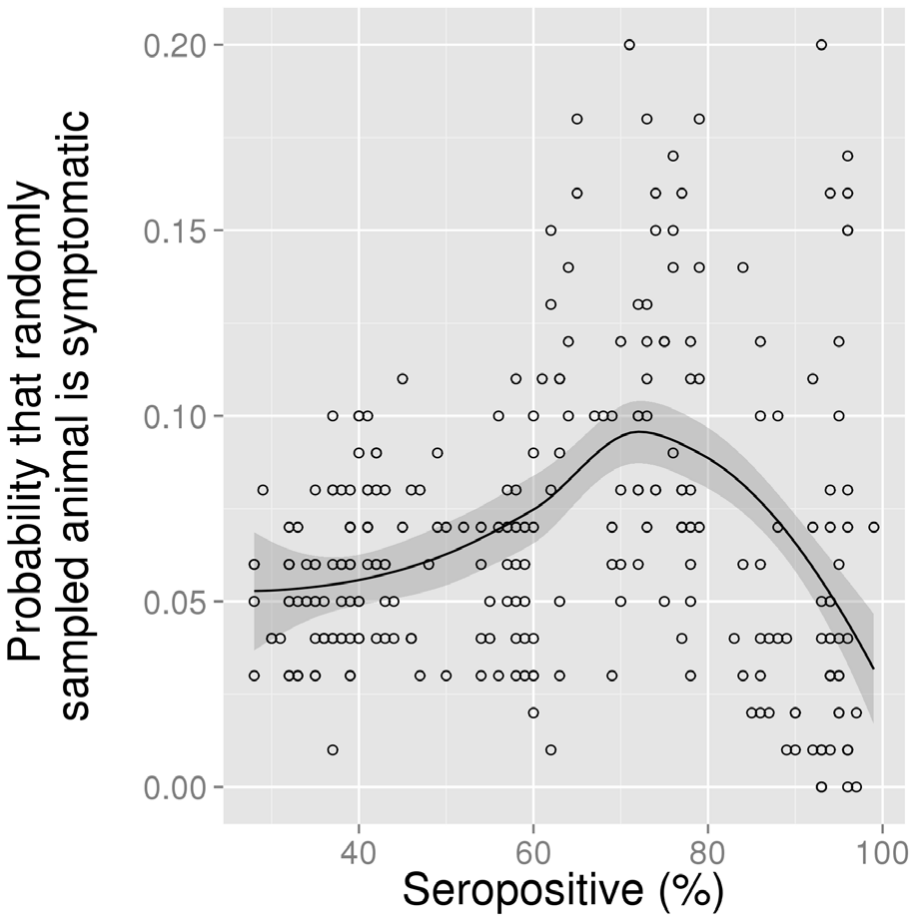
Binomial sampling from the mechanistic model to simulate the likely observed relationship between seroprevalence and case reporting. The mechanistic transmission model was run at a range of transmission rates restricted to between *β* = 10^−1.4^ and *β* = 10^−0.4^ (log scale, step size = 10^−0.2^). Based on equilibria for model states, the probabilities of an animal being seropositive and of being symptomatically infected were calculated. For each of 100 sample sets, a binomial trial was performed 100 times (reflecting 100 animals), with the relevant probability (seropositive or symptomatically infected). Finally, a further subsampling was performed to ensure that 50 data points were used per seroprevalence range (20–40%, 40–60%, 60–80%, 80–100%) in plotting the relationship, in order to avoid the high seroprevalence bias introduced by varying the transmission rate on a log scale. Open circles represent the values for seroprevalence and the probability of symptomatic infection at a single representative sampling of 100 animals at each of the transmission rates tested. The solid black line is a smooth estimate (loess, span = 0.8) of the non-linear relationship between these two variables, based on the complete set of 100 samples. The shaded area around the line is the 95% CI based on the standard errors of the locally weighted least squares regression using the t-based approximation.

Binomial sampling from the mechanistic model at the system equilibrium was performed to generate an example of the likely observed relationship between seroprevalence and disease incidence assuming the model mechanism is in operation. This relationship is shown in figure 6 and is qualitatively comparable to the empirical data shown in figure 4. The two plots differ in their y-axis with the empirical data measuring the proportion of county-years reporting and the model sample measuring the probability that a randomly sampled animal would be symptomatic.

Given the uncertainty in the duration of immunity, we analyzed model predictions in which the duration takes extreme values (along with the empirically-motivated intermediate value–Fig. 4). This extra analysis demonstrates that the main results are not affected by the choice of the parameter controlling duration of time spent in resistant classes. For very short duration of immunity (i.e. interpreting antibody presence in the empirical data of Stallknecht et al. [9] as evidence of re-exposure) we see that there is still a disconnection between transmission intensity and symptomatic cases (Fig. S1). Similarly for long duration of immunity (essentially life-long) the same pattern is observed (Fig. S2).

## Discussion

The concept of enzootic stability has been in the literature for some time [1]. Probable instances have been documented in tick-borne diseases going back several decades [19], [20] and a wide range of human and animal diseases have been identified that satisfy the criteria for its occurrence [2]. However, identifying a system for which there is regional variation in transmission rate (a condition for enzootic stability to manifest) along with estimates of seroprevalence, case prevalence and data to parameterize a transmission model has impeded a rigorous demonstration in nature. It is notable that this demonstration occurs in a vector-borne disease where the *Culicoides* vectors are known to have several ecological requirements. Observational studies have noted that larvae of *Culicoides* spp. are abundant where soils border rivers and streams [21] and consequently have the potential to flourish during droughts as water recedes to create this ideal wet mud habitat. In a regional study of HD, factors including wind speed and rainfall were also predictive, suggesting a high-dimensional ecological niche for the vector [22]. Further, overwintering periods of the vector are likely to vary latitudinally creating spatial variation in the potential for year-round transmission in the host-vector cycle. Other arthropod-borne infectious diseases may also have similar transmission gradients across a landscape. More generally, population density, treatment level and interactions with other diseases are potential reasons for regional variation in transmission [16]. Consequently, the prerequisite variation in transmission needed to create distinct transmission and disease hotspots is likely to operate in a wide range of infectious disease systems.

Coleman et al. [2] highlight several diseases that satisfy the criteria for endemic or enzootic stability, many of which have direct transmission mechanisms. Among the human diseases they consider, polio is an illuminating example where the transmission gradient could also be temporal, rather than spatial [23]. Over time, transmission is likely to have been decreasing in many places; in the original high-transmission scenario there were both many surviving females passing antibodies maternally, and exposure occurring at a young age, when maternal antibodies are still protective. In the early post-industrialization regime, general sanitation improvements resulted in fewer females to pass maternal antibodies, and exposure occurring later in life when such antibodies are no longer protective. Consequently, there were reports of increased outbreaks [23]. Finally, in the vaccine-era, we have seen polio eradicated regionally with hope of global eradication [24]. Historic polio data does not contain detailed information such as age-specific case:infection ratios [23] making it hard to establish a full mechanistic description. Also, the change in transmission intensity is anthropogenic versus an ecological gradient. However, the mechanistic model developed here applies to variation in transmission that is expressed either spatially or temporally, and contributes to understanding maintenance mechanisms in this specific wildlife disease (HD) as well as forming a framework to help identify cryptic transmission hotspots more generally.

A key difference that often exists between human and animal diseases is the quality of the surveillance data. In the HD system, surveillance data is not at the level of individual deer; rather each county within a state is recorded for presence/absence of disease each year. This level of aggregation makes any analysis of such data subject to the modifiable areal unit problem [25], [26], meaning that conclusions depend on the particular choice of units of aggregation–the ideal solution, a true multiscale analysis, is not possible. This makes it especially important to explore opportunities to connect regional presence/absence reporting of wildlife diseases with the suite of population modeling techniques that aim to infer mechanistic processes from data. Under the plausible assumption that a greater number of symptomatic deer will increase the probability of reporting in a county, we are able to connect the data with the model, showing that regions with the highest reporting of cases are those where seroprevalence (and consequently transmission intensity) are intermediate.

Generally, the results of the mechanistic model are in good agreement with the data connecting seroprevalence to the frequency of case reporting. Quantitatively, there is some discrepancy between the exact value of seroprevalence that is associated with the highest probability of disease reporting. Data suggests this occurs at a seroprevalence value around 50% whereas the model indicates 70%. A possible explanation for this lies in the observation that some deer appear to have innate resistance to epizootic hemorrhagic disease [27]. Although experimental infection caused similar viremia profiles in all animals, resistant groups had mild or undetectable disease. This is a mechanism apart from antibody-mediated protection and depending on the proportion of such resistant deer in a population, could lead to a lower than expected value of seroprevalence that corresponds with maximal case reporting. The model's unimodal relationship between transmission rate and cumulative incidence does not occur in the closely related SIR models [16]. Consequently, we can conclude that it is due to the addition of *A* and *P* epidemiological classes, which are an expression of the empirically-motivated criteria for enzootic/endemic stability [2].

Awareness of enzootic and endemic stability enhances our explanatory and predictive power in the context of disease development at the population level, including inference of key parameters. Regions with low case reporting could have the potential to act as cryptic sources, supplying infection to areas where transmission is lower, yet case reporting is relatively high. Consequently, both surveillance and control options can be improved by understanding that many infectious disease systems do not follow the axiom that the number of cases is positively associated with transmission rate. Indeed, related but distinct mechanisms have been put forward to explain dengue hemorrhagic fever patterns [28] highlighting that the relationship between outbreak data and transmission processes may commonly be more complex than often acknowledged.

Ecological niche modeling is a growing area in infectious disease research, particularly for vector-borne diseases [29]. While using presence and absence of reported disease may help establish a vector's ecological niche in situations where transmission equals disease, caution must be taken when there is evidence of a more complicated relationship between transmission and disease occurrence, as is the case here. Seroprevalence surveys could help establish regions with cryptic transmission in these cases.

In relation to disease control, operation of mechanisms similar to those exhibited in the HD system introduce the ethically-charged issue of whether it might be desirable to maintain (or possibly enhance) transmission intensity in the hope of reducing disease burden. When variation in transmission is expressed along a spatial ecological gradient, this could have the unintended consequence of increasing the capability of cryptic source regions to seed outbreaks elsewhere. At the very least, such mechanisms serve as a warning that efforts to control disease could also have the unintended effect of increasing disease burden if transmission intensity is not weakened into a regime of low disease occurrence.

## Supporting Information

Figure S1 **Model predictions under a scenario of short duration of immunity.** As Figure 2 but with waning immunity rate (ω) chosen to reflect a mean duration of protection of 6 months.
(DOCX)

Figure S2 **Model predictions under a scenario of long duration of immunity.** As Figure 2 but with waning immunity rate (ω) chosen to reflect a mean duration of protection equal to the mean deer lifespan.
(DOCX)

Equations S1 **System of ordinary differential equations (notation explained in main text).**
(DOCX)
