# Supplementary material for: When More Transmission Equals Less Disease: Reconciling the Disconnect between Disease Hotspots and Parasite Transmission
Source: PLoS One. 2013 Apr 8;8(4):e61501. doi: 10.1371/journal.pone.0061501 (PMC3620270; doi:10.1371/journal.pone.0061501)
Supplement: Figure S1 — Model predictions under a scenario of short duration of immunity. As Figure 2 but with waning immunity rate (ω) chosen to reflect a mean duration of protection of 6 months. (DOCX) [file pone.0061501.s001.docx]

Fig. S1 - Supplementary Material for Park et al. “**When more transmission equals less disease: reconciling the disconnect between disease hotspots and parasite transmission**”


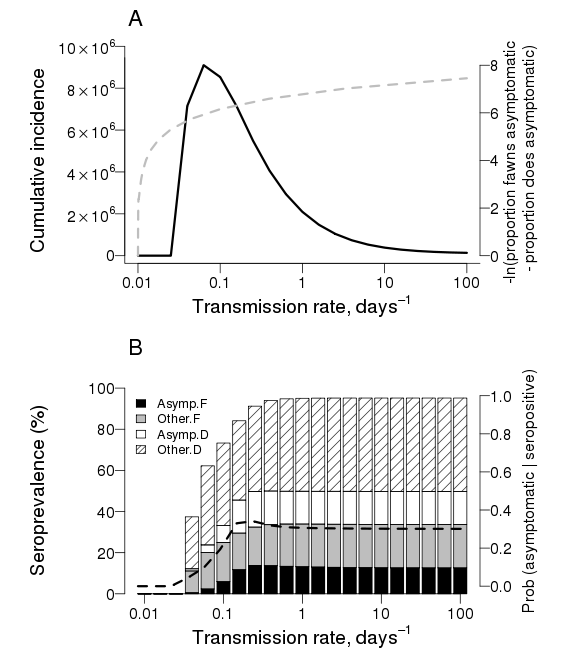


**Figure S1. Model predictions under a scenario of short duration of immunity.**

As Figure 2 but with waning immunity rate (ω) chosen to reflect a mean duration of protection of 6 months.
