# Supplementary material for: When More Transmission Equals Less Disease: Reconciling the Disconnect between Disease Hotspots and Parasite Transmission
Source: PLoS One. 2013 Apr 8;8(4):e61501. doi: 10.1371/journal.pone.0061501 (PMC3620270; doi:10.1371/journal.pone.0061501)
Supplement: Equations S1 — System of ordinary differential equations (notation explained in main text). (DOCX) [file pone.0061501.s003.docx]

Model equations S1 - Supplementary Material for Park et al. “**When more transmission equals less disease: reconciling the disconnect between disease hotspots and parasite transmission**” - 1^st^ page = Fawns

2^nd^ page = Does
